# Critical and Glassy Dualism in Glass-Forming E7 Nematogenic Mixture and Fullerene C_60_ Nanocolloids

**DOI:** 10.3390/nano16171123

**Published:** 2026-09-07

**Authors:** Aleksandra Drozd-Rzoska, Mateusz Kotowski, Jakub Kalabiński, Tushar Rajvanshi, Sylwester J. Rzoska

**Affiliations:** Institute of High Pressure Physics of the Polish Academy of Sciences, ul. Sokołowska 29/37, 01-142 Warsaw, Poland

**Keywords:** liquid crystals, nanoparticles, fullerenes, broadband dielectric spectroscopy, phase transitions, critical effects, glass transition

## Abstract

The report presents the results of broadband dielectric spectroscopy (BDS) studies in bulk nanocolloids: E7 liquid crystalline (LC) mixture plus C_60_ fullerene nanoparticles. BDS spectra for 260 temperatures from the isotropic liquid (I) phase at ~360 K to the nematic (N) phase at the glass temperature $T_{g}\;\sim \;220\;K$ were tested. The analysis focused on pretransitional critical-like features and complex glassy dynamics, highlighting their interplay and dominance. This is associated with pretransitional fluctuations, which impact the nematic phase even 90 K below the I-N transition. On approaching $T_{g}$, strong previtreous changes detected via dielectric constant and the loss curve maximum appear, starting at $T_{g}+30\;K$. The critical-like behavior on $T\to T_{g}$ was observed also for the parameter describing the distribution of relaxation times. For the three main relaxation times in the long-range nematic phase, the optimal portrayal via the new *Critical* and *Activated* equation is evidenced. The derivative-based test of coupling/decoupling between translational and orientational processes reveals strong decoupling with the fractional exponent F<1 below the I-N transition and related to F<1 above $T_{g}$. Notable is the permanent ‘parallel’ orientation of LC molecules by C_60_ fullerene nanoparticles, which can be significant for applications.

## 1. Introduction

Nematogenic mixture E7 is a unique liquid crystalline (LC) material, originally designed to achieve a nematic phase over a very wide temperature range, from ~65 °C down to −50 °C. It is composed of rod-like LC materials with a relatively large permanent dipole moment parallel to the long molecular axis [1,2]. These features led to enormous success in applications, including widespread use in displays, smart windows, soft robotics, or laser optics [2,3,4,5,6]. There are several legitimate cognitive challenges where E7-related research may be of significant importance:

- E7 is one of the most important canonical materials in the Physics of Liquid Crystals [2,3] and also in Critical Phenomena Physics [7,8]. The latter is particularly related to pretransitional effects with ‘critical’ characteristics in the wide region surrounding the isotropic liquid (I)–nematic (N) phase transition, despite its discontinuity. This phenomenon inspired the Landau–de Gennes (LdG) model, which was particularly important in awarding Pierre G. de Gennes the Nobel Prize in 1991 [9]. The governing feature is related to multimolecular fluctuations, whose magnitude increases ‘critically’ as the phase transition approaches. This phenomenon and the LdG model are discussed in a recent publication by the authors, which also highlights remaining challenges. The latter includes the scope of critical fluctuations’ influence in the nematic phase [8]. E7 research offers unique opportunities here because of the extreme range of the nematic phase.
- E7 mixture is also a significant material for studies of glass transition [1,2,3,4,5,6,7,8,9,10,11,12], a cognitive mystery considered among the grand challenges of 21st-century science [13,14,15,16,17,18,19,20,21,22,23,24]. Supercooling and vitrification are particularly important because they are associated solely with the liquid crystalline nematic phase and the molecule’s uniaxial symmetry. The latter has recently been shown to be essential for the manifestation of some universal previtreous effects.
- The properties of liquid crystalline materials are strongly influenced by external (exogenous) factors, such as pressure or a strong or even moderate electric field. Equally important are endogenous (‘internal’) factors, such as admixtures of non-mesogenic solvents: even tiny amounts of which can strongly change transition temperatures. Nanoparticles are also among these endogenous factors [25,26,27,28,29,30,31,32,33,34,35,36,37,38,39,40,41,42,43,44,45,46,47,48,49,50,51,52,53,54,55,56]. It may be surprising that the frustrating impact of nanoparticles on critical-type pretransitional effects and previtreous phenomena remains with still-limited evidence [57,58,59,60].

This report aims to address these cognitive gaps through broadband dielectric spectroscopy (BDS) studies of E7 and related nanocolloids with C_60_ fullerene nanoparticles.

However, first, the authors want to recall some aspects related to the phenomena mentioned above. The basics of critical phenomena are widely known, as this topic is among the grand successes of 20th-century physics [7,8,9]. Less well-known are the specifics of previtreous dynamics, even though the problem is considered a major challenge of 21st-century science [13,14,15,16,17,18,19,20,21,22,23,24].

The hallmark of the previtreous phenomena is a set of universalistic features extending well above the glass temperature $T>T_{g}$ [14]. One can recall: (*i*) non-Arrhenius changes in primary relaxation time (*τ*, *τ_α_*), viscosity (*η*), DC electric conductivity (*σ*)… (*ii*) decoupling between translational and orientational dynamics, (*iii*) the non-Debye distribution of the primary relaxation time, (*iv*) the dynamic crossover, often linked to the ‘magic’ time scale $\tau \left(T_{B}\right)=10^{-7\pm 1\;}s$, (*v*) the secondary relaxation emerging for $T<T_{B}$, and related to the time scale $\tau_{\beta }<\tau_{\alpha }$—usually associated with the Arrhenius pattern of temperature changes, (*vi*) ‘~universality high-frequency distribution parameter $n\left(T\to T_{g}\right)$ →1/2, of the primary relaxation time, (*vii*) common presentation of primary relaxation time via the normalized scale in the Angell plot.

This list of ‘universalities’ is far from complete, but it shows the dominance of dynamics-related features. No ultimate model still coherently addresses these checkpoint-validating properties [14,15,16,17,18,19,20,21,22,23,24].

The strong influence of nanoparticles on the properties of LC reference materials has remained the subject of numerous intensive studies in recent decades [25,26,27,28,29,30,31,32,33,34,35,36,37,38,39,40,41,42,43,44,45,46,47,48,49,50,51,52,53,54,55,56,57,58,59,60,61]. This is largely driven by the expectation of obtaining composite materials with innovative features that enhance the applications mentioned above [51,52,53].

Surprisingly, studies focused on the previtreous features of LC + nanoparticles systems are very limited. This includes E7-based nanocolloids, despite the fact that E7 nematogenic mixture is particularly important for glass transition studies. Probably, the only explicitly focused studies have only recently been reported for E7 + BaTiO_3_ nanoparticles (diameter 2r=50 nm, paraelectric) nanocolloids [61].

This report presents the results of broadband dielectric spectroscopy (BDS) studies on E7 + C_60_ fullerene (d=0.7 nm) nanocolloids, focusing on previtreous and critical properties. We implement extended analysis when comparing with ref. [61], which highlights exceptional features significant for both fundamental insight and applications.

## 2. Materials and Methods

E7 is the eutectic mixture composed of rod-like cyanobiphenyl and cyanoterphenol components at a specific composition, namely (**1**) 4-cyano-4’-n-pentyl-biphenyl (5CB, 51%), (**2**) 4-cyano-4’-n-heptyl-biphenyl (7CB, 25%), (**3**) 4-cyano-4’-n-oxyoctyl-biphenyl (8OCB, 16%), and (**4**) 4-cyano-4’’-n-pentyl-p-terphenyl (5CT, 8%) [1,2]. E7 nematogenic mixture was purchased from Synthon Chemicals GmbH & Co. KG (Bitterfeld-Wolfen, Germany) at the highest available quality. Prior to measurements, it was degassed and purified in subsequent and repeated steps: (*i*) solidification by freezing, supported by liquid nitrogen, (*ii*) removal of air and vapors via a vacuum pump, (*iii*) heating up to ca. 90 °C i.e., deeply into the isotropic liquid state. The tested samples exhibit the following mesomorphism: solid glass—(220 ± 5 K)—nematic—(332.9 K)—isotropic liquid—in agreement with referenced results [1,2,3,4]. The transition to the glass state is ‘diffused’, as indicated in the description.

For each LC compound in E7 mixture, there is a notable permanent dipole moment (≈5 Debye), approximately parallel to the long molecular axis. The length of the molecules in E7 mixture ranges from ~1.8 nm (5CB) to ~2.2 nm (8OCB) [1,2,3,4].

Fullerene C_60_ nanoparticles (diameter 2r≈0.7 nm) were purchased from Sigma-Aldrich, Inc. (St. Louis, MO, USA). From dielectric tests in dilute solution, the dielectric constant of such fullerenes was estimated as ε≈3.8 [58]. Nanocolloids were prepared using relatively large volumes of E7, $\sim 5{\;cm}^{3}$, in sample preparation to estimate nanoparticles (NPs) concentration (mass%) reliably. Nanocolloidal samples were sonicated for ca 1 h prior to filling the measurement module. Earlier studies by the authors [57,58,59,60,61] showed that in bulk nematogenic and smectogenic LC compounds, sedimentation and/or self-assembly of nanoparticles appear for mass concentrations x greater than 1%, which explains the concentration range tested in the given report. It was tested optically, using samples placed between two glass plates under polarization microscopy and by dielectric constant tests with different arrangements of the flat-parallel capacitor. A common practice in such nanocolloids is to add macromolecules, which, when anchored to the nanoparticles, stabilize the nanocolloid against sedimentation or self-assembly. However, these macromolecules can dominate the recorded broadband dielectric spectroscopy (BDS) [62] spectra, making it impossible to reliably observe the crucial mutual influence between the “liquid crystal matrix” and nanoparticles. The working conditions in this area prevent this “parasitic” factor from being present in BDS studies.

Studies focused on small concentrations of nanoparticles NPs, following previous findings of refs., indicating that this can be a region of surprisingly strong influence on the system compared to the native E7. For the tested concentration range (x≤1%), no sedimentation occurs, allowing us to avoid a supplementary macromolecular compound that is often used to stabilize the colloid [57,58,59,60,61]. However, the presence of such supplements complicates the analysis of BDS spectra due to a very strong ‘parasitic’ effect from these macromolecules, both attached to nanoparticles and ‘free’.

**Figure 1:**
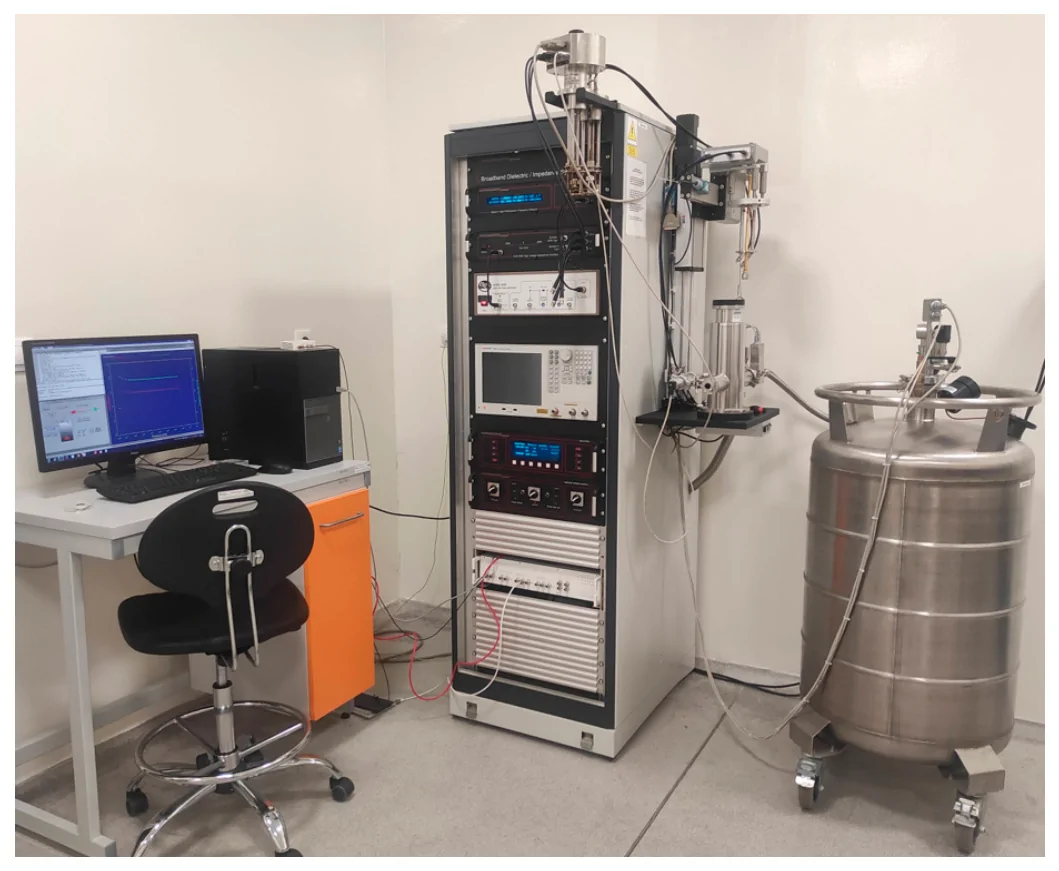
The photo shows the applied Novocontrol BDS impedance analyzer with the temperature control unit and system control desk. The facility is in the X-PressMatter Lab IHPP PAS [https://young4softmatter.pl/].

Broadband dielectric spectroscopy (BDS) is essential for liquid crystalline materials and their colloid-based systems because of their enormous sensitivity to electric fields, which underlies numerous applications [1,2,3,4,8,57,58,59,60,61,62]. BDS studies were carried out using a Novocontrol Technologies GmbH & Co. KG (Montabaur, Germany) impedance analyzer, coupled with the extended Quattro Novocontrol temperature control unit. Figure 1 shows the facility.

Tested samples were placed in a flat-parallel capacitor, with d=0.3 mm gap, and 2r=20 mm diameter, made from Invar. The applied voltage for measuring the electric field was U=1 V, which enabled a permanent 6-digit resolution during measurements and ensured a weak, ‘negligible ’ intensity of the electric field.

The authors stress the resolution because all basic characterizations discussed in the given report were taken directly from detected impedance spectra, considered via the complex dielectric permittivity, as presented in Section 3 below. Such analysis is possible when significant phenomena influencing BDS spectra are well manifested. They are: the dielectric constant, the primary loss curve maximum ($\varepsilon^{\prime \prime }\left(f\right)$), DC electric conductivity… as also stressed below. Here, the error is less than the size of the ‘points’ in subsequent figures presenting data. For supporting results, namely the derivative analysis, the error is indicated by the scatter of the transformed data, which is minimal for this type of analysis. Values of all fitted parameters obtained from the scaling analysis are given with error defined as three standard deviations, related to ~0.3% of data lying outside the corridors defined by this error.

All measurements were repeated twice, on cooling and heating. The first, basic test was the ‘dense’ scan, where the step between subsequent measurements changes from ~1 K (remote phase/glass transitions) to ~0.2 K in their immediate surroundings. This monitoring can last up to 30 h to reach the required temperature stabilization level, which is only monitored by the Quattro system. The second control scan involves ca. 20 selected temperatures to test overlapping results with the basic, ‘dense’ scan results. If overlap occurs, no other tests are carried out. If not, the whole process is repeated. This was not the case in the presented studies. Note that the addition of a ‘reliability test’ is the comparison with earlier available reference results. This was possible for the dielectric constant in the isotropic liquid phase, which is sufficient to test the detected values across the whole BDS spectrum.

For the results presented below, the disturbing impact of ionic conductivity from ‘ionic contaminations’, the coupled intrinsic dielectric relaxation, or the anchoring effect introduced by capacitor-plate micro/nano-constraints is not important. First, the studies were carried out for bulk samples using a capacitor with a macro-gap. For the remaining issues, the spectrum presented in Section 3 below shows that they do not influence the static domain associated with the dielectric constant or the characterization of the loss curves discussed.

## 3. Results

**Figure 2:**
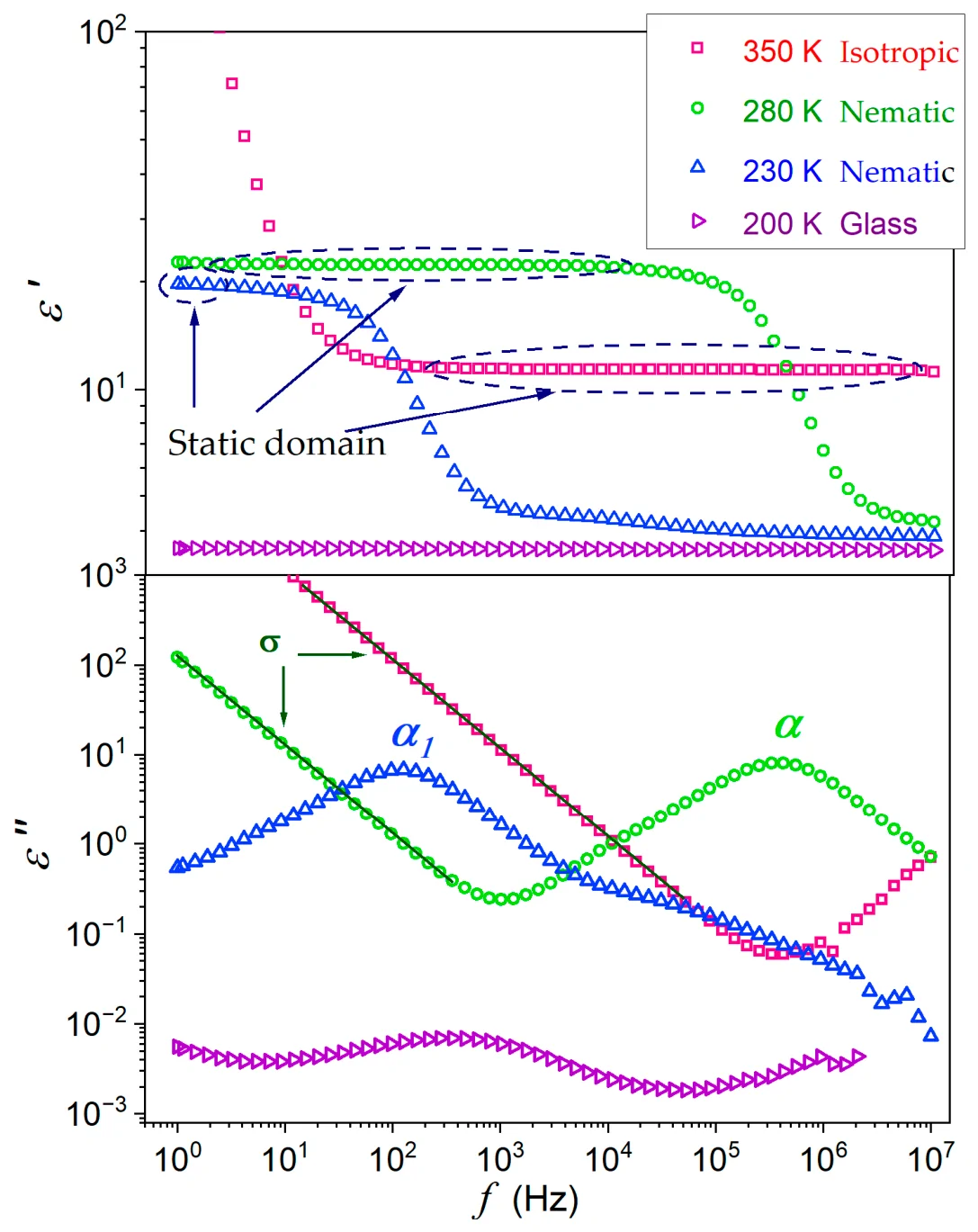
Selected characteristic spectra of the real and imaginary components of dielectric permittivity in subsequent phase/states of E7 + C_60_ fullerene nanoparticles nanocolloids (x=0.5%). The spectrum for pure E7 is given in ref. [61].

Figure 2 shows the representative spectra of the real and imaginary parts of dielectric permittivity in E7 + NPs (NanoParticles: C_60_ fullerene) nanocolloid. They are selected from spectra measured over 260 temperatures. Characteristic features and frequency domains are indicated. Notable is the horizontal static domain for the real part of dielectric permittivity $\varepsilon^{\prime }\left(f\right)$, defining the dielectric constant. As the frequency increases above the static domain, the impact of the permanent dipole moment diminishes, and $\varepsilon^{\prime }\left(f\right)$ significantly decreases. This is the High-Frequency (HF) domain. On decreasing the frequency below the static domain $\varepsilon^{\prime }\left(f\right)$ values strongly rise. It is the low-frequency (LF) domain. This exceptional rise in low-frequency (LF) domain is most often heuristically explained by the influence of residual ionic ‘contaminations’. For the authors, it can also be due to local translational shifts in the basic molecules that constitute the system. Such an origin supports translational–orientational coupling/decoupling that links the LF and HF domains, as shown below for systems tested in the given report.

Namely, in the spectrum presented in Figure 2, DC electric conductivity is estimated by $\sigma =\omega \varepsilon^{\prime \prime }\left(f\right)$ in the low-frequency domain. Distortions from such a pattern can occur in the low-frequency region and may suggest effects related to electrode polarization, but this is not the case with the results presented in Figure 2.

The primary (*alpha*) relaxation time is determined from the primary loss curve peak $\varepsilon_{peak}^{\prime \prime }\left(f\right)$, namely: $\tau =1/\omega f_{peak}$. This is the high-frequency part of $\varepsilon^{\prime \prime }\left(f\right)$ spectrum. The low-frequency part of the same $\varepsilon^{\prime \prime }\left(f\right)$ determined the DC electric conductivity as mentioned above. Both properties are coupled by the Debye–Stokes–Einstein (DSE) relation σ×τ=const, or its fractional (fDSE) extension $\sigma \times \tau^{F}=const$ [59]. This is the coupling between orientational and translational motions, using data $\varepsilon^{\prime \prime }\left(f\right)$ taken from the same spectrum. This is easy to understand if the phenomenon relates to the orientational and translational movements of the same basic molecule. For the authors, the often-suggested link of DC electric conductivity to unspecified ‘ionic contaminations’, i.e., species essentially different from ‘base molecules’, cannot explain the DSE or fDSE behavior that is well validated experimentally.

The real part of dielectric permittivity is associated with the relative change in electric capacitance, $\varepsilon^{\prime }\left(f\right)=C\left(f\right)/C_{0}$, where $C_{0}$ is the reference capacitance of the capacitor with no sample. This is related to the upper part of Figure 2. The lower part of Figure 2 shows the coupled behavior of the imaginary part of dielectric permittivity $\varepsilon^{\prime \prime }\left(f\right)=1/\omega R\left(f\right)C\left(f\right)$, where ω=2πf. For $\varepsilon^{\prime \prime }\left(f\right)$ spectrum loss curves peaks reflecting the leading relaxation process, particularly associated with the orientation of permanent dipole moments, appear. The peak (maximum) of the loss curve is related to the relaxation time characterizing the given process, namely: $\tau =1/\omega_{peak}$, and $\omega_{peak}=2\pi f_{peak}$ [62]. Loss curves ‘wings’ enable insight into the distribution of relaxation times. Jonsher [63,64] introduced their following ‘universal’ empirical scaling characterization:

(1) $$\varepsilon^{\prime \prime }\left(f\right)\propto f^{m}\;\;\;\;\;\Rightarrow \;\;\;log\varepsilon^{\prime \prime }\left(f\right)\propto m\times logf\;\;\;\;\;\;\;\;\;\mathrm{for}\;f<f_{peak}$$

(2) $$\varepsilon^{\prime \prime }\left(f\right)\propto f^{-n}\;\;\;\;\Rightarrow \;\;\;log\varepsilon^{\prime \prime }\left(f\right)\propto -n\times logf\;\;\;\;\;\;\;\;\;\mathrm{for}\;f>f_{peak}$$

where $f_{peak}$ is the frequency related to the loss curve maximum, and 0<m,n<1 are parameters characterizing the low- and high-frequency related distributions of relaxation time.

Equations (1) and (2) can be applied for the direct estimation of the distribution parameters via the following transformation of empirical data [65,66,67]: $dlog\varepsilon^{\prime \prime }\left(f\right)\left(f\right)/dlogf=m$—for <$f_{peak}$ and $dlog\varepsilon^{\prime \prime }\left(f\right)\left(f\right)/dlogf=-n$ for >$f_{peak}$, also allowing us to estimate the loss curve peak frequency: $dlog\varepsilon^{\prime \prime }\left(f=f_{peak}\right)/dlogf=0$.

Formally, Jonscher scaling [63,64] is a consequence of the behavior near the inflection point, where rapid functional growth transforms into a slower one.

An alternative path of analyzing loss curves is direct portrayal via the Havriliak–Negami (HN) equation [62,68,69]:

(3) $$\varepsilon^{*}\left(\omega \right)=\varepsilon_{\infty }+\frac{\Delta \varepsilon }{{\left[1+{\left(i\omega \tau \right)}^{a}\right]}^{b}}+\frac{\sigma_{DC}}{i\varepsilon_{0}\omega^{\Phi }}$$

where ω=2πf, $\varepsilon_{\infty }$ is the ‘infinite frequency’ terminal values related to the sum of atomic and electronic polarizabilities, *Φ* is related to the distortion from the DC conductivity limit related Φ=1. Parameters a,b<1 describe the distribution of relaxation times; a,b=1 are for the single-relaxation-time Debye model [62].

Distribution-related parameters in Jonscher’s and HN scalings are related, namely: m=b and n=ab. The analysis employing HN Equation (3) requires multi-parameter nonlinear fitting, and then it is inherently associated with a notable error. Nevertheless, it is the only tool where loss curves overlap, limiting the reliable application of Equations (1) and (2).

When decreasing the frequency below the loss curve manifestation in $\varepsilon^{\prime \prime }\left(f\right)$ spectrum, one enters the part of the spectrum enabling the estimation of the DC electric, $\sigma^{\prime }\left(f\right)={\omega \varepsilon }^{\prime \prime }\left(f\right)$ →$\sigma_{DC}=\sigma =const$. It is related to the pattern $\varepsilon^{\prime \prime }\left(f\right)\propto f^{-\phi }$, with ϕ=1 [60,61], as visible in Figure 2.

To obtain insight into temperature changes in properties influencing BDS spectra, we tested over 260 temperatures for the isotropic and nematic phases, down to the vicinity of the glass temperature. The dielectric constant is historically the first and remains an essential characterization of dielectric properties. Most often, it is determined by following $\varepsilon^{\prime }\left(f,T\right)$ changes in frequencies f=10 kHz or f=100 kHz. However, this is not the case for glass-forming liquids, in which a significant shift toward lower frequencies occurs as the glass temperature is approached. This is well-illustrated by the shift in the primary relaxation time from $\tau \sim 10^{-8}\;s$ near I-N transition to $\tau \approx 10^{2}\;s$ at the glass temperature $T_{g}$. Hence, in the given report, dielectric constant has been taken at frequencies in the middle of the static domain, i.e., at frequencies that naturally shift to lower frequencies on cooling towards $T_{g}$.

**Figure 3:**
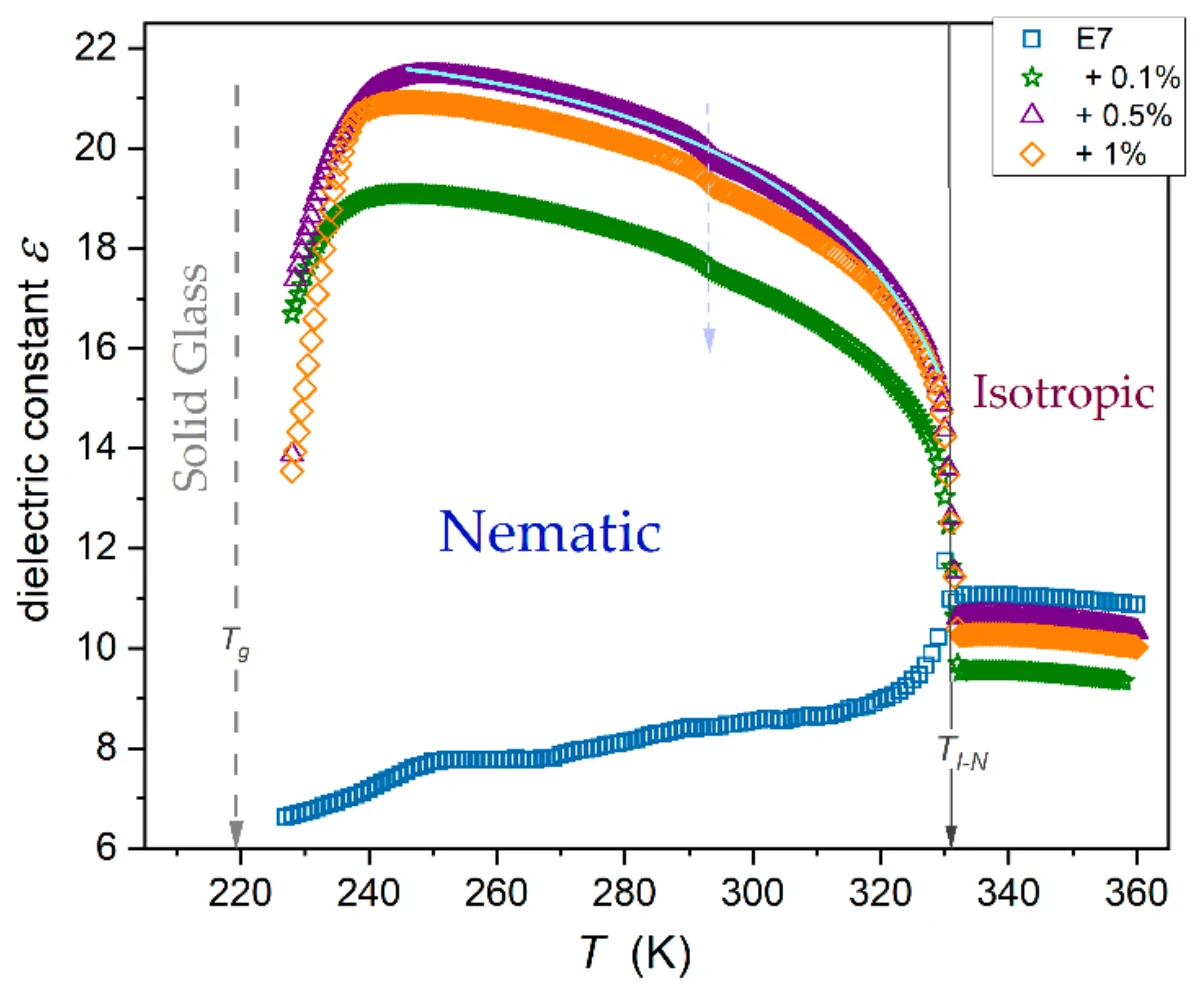
Changes in dielectric constant in E7 and related nanocolloids with C_60_ fullerene nanoparticles in the isotropic liquid and nematic phases, down to the glass temperature. The scaling of data in the nematic phase for x=0.5% is related to Equation (11) with the following parameters: $b_{1}=12.4$, $b_{2}=-0.48$, $b_{3}=1.9$, $b_{4}=-0.08,$ and $T^{**}=334.2\;K$.

**Figure 4:**
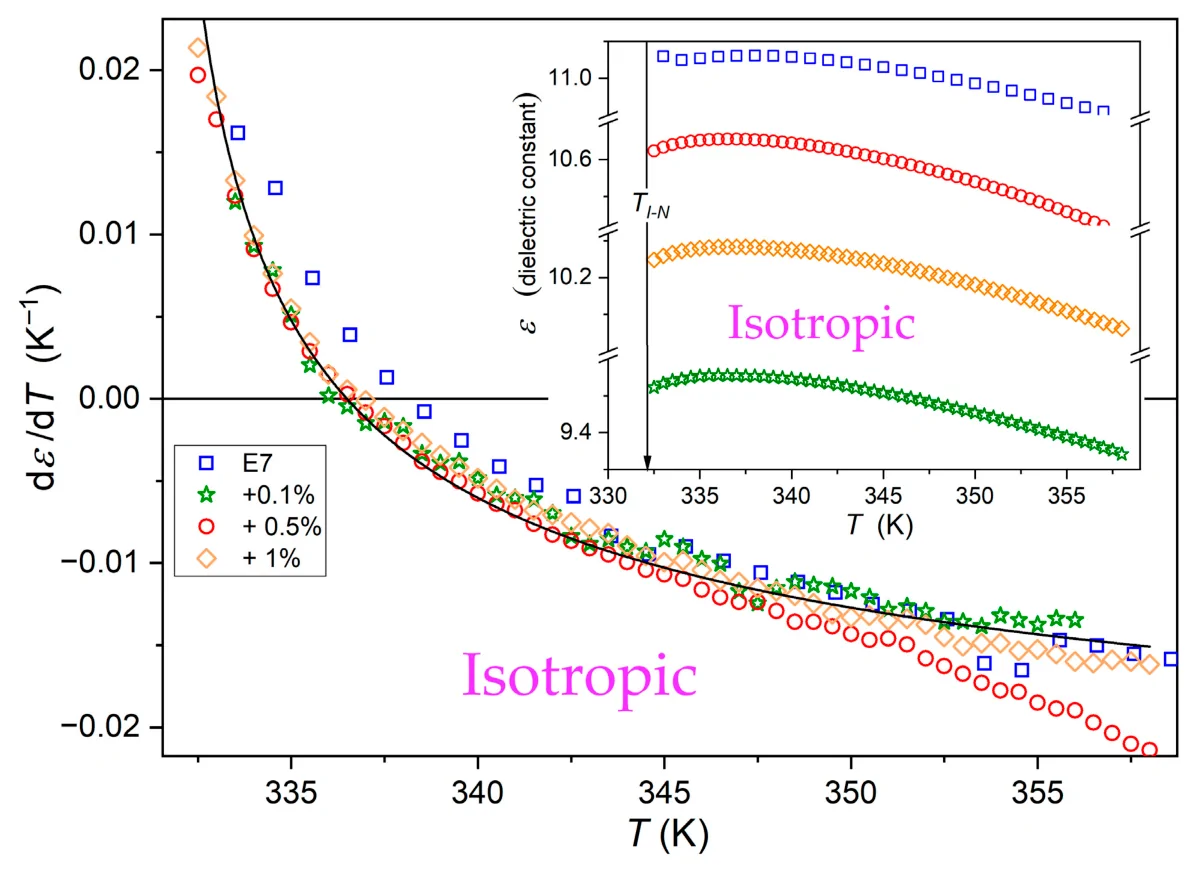
Focused insight into the behavior of the dielectric constant in the isotropic liquid phase of E7 + C_60_ fullerene nanocolloids, shown in the inset. The central part of the plot shows the derivative of the data from the inset to visualize the critical-like anomaly. Note the scaling via Equation (7) and the link to Equation (6).

Figure 3 shows dielectric constant temperature changes in the isotropic and nematic phases of E7 and related nanocolloids. Figure 4 presents a focused view of the isotropic liquid phase.

The isotropic liquid–nematic (I-N) phase transition is a classic example of a weakly discontinuous phase transition, assisted by long-range critical-type pretransitional effects. Drozd-Rzoska et al. [8,70,71] developed a model for scaling dielectric-constant changes, drawing on Critical-Phenomena Physics. As one approaches a continuous phase transition, long-range pretransitional effects arise from pretransitional fluctuations whose local symmetry is linked to the next approaching phase. Their lifetime ($\tau_{fl.}$) and size (correlation length, ξ) increase infinitely as they approach the critical singularity. In the given case, it can be associated with the extrapolated critical-like or pseudospinodal-type temperature $T^{*}$ [7,8,72]:

(4) $$\tau_{fl.}\left(T\right)=\tau_{0}^{fl.}{\left(T-T^{*}\right)}^{-zv}$$

(5) $$\xi \left(T\right)=\xi_{0}{\left(T-T^{*}\right)}^{-v}$$

where $T>T_{I-N}$, is the extrapolated singular $T^{*}=T_{I-N}-{\Delta T}^{*}$, ${\Delta T}^{*}$ is the metric of I-N transition singularity, the correlation length exponent is ν=1/2 (in the given case), and z=2 is the dynamic exponent for the non-conserved order parameter.

Pretransitional, critical-like fluctuations are associated with the basic characterization of the next approaching phase. In the isotropic liquid phase, on approaching the I-N transition, it involves local prenematic ordering of rod-like molecules. For rod molecules with the permanent dipole moment approximately parallel to the long molecular axis, as in E7, this leads to their antiparallel arrangement. Consequently, within prenematic fluctuations, the contribution from permanent dipole moments to the dielectric constant is negligible, and thus $\varepsilon \left(fluct.\right)\ll \varepsilon \left(surrounding\right)$. The rise in the correlation length on cooling causes the volume occupied by prenematic fluctuations to become even stronger, namely: $V_{fluct.}\left(T\right)=\xi^{3}\left(T\right)\propto {\left(T-T^{*}\right)}^{-3/2}$. Following these, one can expect the dominance of the volume occupied by pretransitional fluctuations at some temperature above $T_{I-N}$ and consequently the decrease in $\varepsilon (T{\to T}_{I-N})$ finally takes place on cooling.

The model analysis developed in refs. [8,70,71] showed that it leads to the following relation for dielectric constant pretransitional scaling in the isotropic liquid phase:

(6) $$\varepsilon \left(T\right)=\varepsilon^{*}+a\left(T-T^{*}\right)+A{\left(T-T^{*}\right)}^{\varphi }$$

where $T^{*}<T_{I-N}$ is the extrapolated continuous phase transition temperature, a,A=const; the exponent φ=1−α, where the exponent α=1/2 is the heat capacity (internal energy) critical exponent.

The portrayal via the above relation is presented in the inset of Figure 4. The effect is more pronounced for the derivative of dielectric constant, as shown in the central part of Figure 4 [8,70,71]:

(7) $$\frac{d\varepsilon }{dT}\propto {\left(T-T^{*}\right)}^{-1/2}$$

where the exponent φ−1=−α=−1/2.

Figure 3 also shows the overlap of pretransitional effects expressed as the derivative of dielectric constant dε/dT for tested nanocolloids. Hence, adding nanoparticles changes only the constant term $\varepsilon^{*}$ in Equation (6). Superior scaling of the pretransitional effect in the isotropic phase via Equations (6) and (7) explicitly validates the dominance of multimolecular prenematic characterization, with critical-type—or, more precisely, pseudospinodal-like—characterization [8,70,71].

For the nematic phase, dielectric constant is most often used for testing the order parameter behavior [7,71]: $S=\varepsilon_{∥}-\varepsilon_{⊥}\propto {\left(T-T^{**}\right)}^{\beta }$, where β is the order parameter critical exponent and $T^{**}>T_{I-N}$ is extrapolated from the nematic phase singular temperature; $\varepsilon_{∥}$ and $\varepsilon_{⊥}$ are dielectric constants for the ‘parallel’ and ‘perpendicular’ arrangement of rod-like molecules with respect to the measurement electric field in the capacitor. The distortions-sensitive analysis explicitly showed the mean-field tricritical (TCP) behavior of the I-N transition, associated with β=1/4 [71].

In bulk samples, the orientation of molecules is realized by the external strong magnetic field of the value ~1–2 Tesla [1,2,7]. For capacitors with a micrometric gap (‘thin layer’) the orientation by special preparation of plates, for instance covering by a polymeric layer, is used [1,2]. However, it introduces side effects that require supplementary model assumptions in the analysis [1,2].

A generally accepted heuristic assumption is that just a few degrees below $T_{I-N}$ both $\varepsilon_{∥}\left(T\right)$ and $\varepsilon_{⊥}\left(T\right)$ follow a ‘parallel pattern’ [1,2]. This leads to the basic way of determining the anisotropy of the dielectric constant $\Delta \varepsilon \left(T\right)=\varepsilon_{∥}\left(T\right)-\varepsilon_{⊥}\left(T\right)\;\to \Delta \varepsilon =\varepsilon_{∥}-\varepsilon_{⊥}=const$ (‘parallel pattern’), one of the most important material characterizations of nematogenic LC materials [1,2]. However, there is a formal problem here: as we show below in the given report and earlier in ref. [71], the ‘parallel pattern’ assumption is incorrect. Namely the scaling of $\varepsilon_{∥}\left(T\right)$ is scaled by the following equation [71]:

(8) $$\Delta \varepsilon \left(T\right)=\varepsilon_{∥}-\varepsilon_{⊥}\approx \varepsilon_{\Delta }^{**}+B{\left(T^{**}-T\right)}^{\beta }$$

However, as shown in ref. [69] one can also consider the anomalous pretransitional behavior of the ‘diameter’ in the nematic phase $\sigma \left(T\right)$:

(9) $$\sigma \left(T\right)=\frac{1}{3}\varepsilon_{∥}+\frac{2}{3}\varepsilon_{⊥}\approx \varepsilon_{\delta }^{**}+D{\left(T^{**}-T\right)}^{1-}+d\left(T^{**}-T\right)$$

where $T<T_{I-N}$, B,d,D=const, $T^{**}$ is the hypothetical continuous transition extrapolated from the nematic phase.

Linking Equations (8) and (9), one obtains relations describing changes in dielectric constant for the ‘perpendicular’ and ‘parallel’ contributions [71]:

(10) $$\varepsilon_{⊥}\left(T\right)\approx a_{1}-a_{2}{\left(T^{**}-T\right)}^{\beta }+a_{3}{\left(T^{**}-T\right)}^{1-\alpha }+a_{4}\left(T^{**}-T\right)$$

(11) $$\varepsilon_{∥}\left(T\right)=b_{1}+b_{2}{\left(T^{**}-T\right)}^{\beta }+b_{3}{\left(T^{**}-T\right)}^{1-\alpha }+b_{4}\left(T^{**}-T\right)$$

where $a_{i}$,$b_{i}=const$ denote empirical parameters related to Equations (8) and (9).

Recalling the evidence indicating the near-tricritical nature of the I-N transition for exponents, one can assume: β=1/4 and α=1/2.

Already in refs. [57,60,61], it was indicated that for selected concentrations of BaTiO_3_ (2r=50 nm) dispersed in nematogenic LC ‘matrix’ (concentration x≤1%) the endogenic, permanent orientation in the nematic phase appears. Consequently, the dielectric constant in non-oriented samples, i.e., without the impact of an external strong ‘oriented’ field, followed the pattern of $\varepsilon_{⊥}\left(T\right)$ or $\varepsilon_{∥}\left(T\right)$, depending on the concentration of nanoparticles.

Figure 3 shows that for E7 + C_60_ fullerene nanocolloids, the dielectric constant in non-oriented samples spontaneously follows the $\varepsilon_{∥}\left(T\right)$ pattern. This parameterization is expressed by Equation (11). The validity of such scaling is shown in Figure 3.

The above pattern of $\varepsilon \left(T\right)$ evolution undergoes a qualitative change from the pattern described by Equation (11) at approximately 25 K above the glass temperature $T_{g}$, manifested via a significant decrease in detected values. Notably, for E7 + BaTiO_3_ nanocolloids, which initially follow the $\varepsilon_{⊥}\left(T\right)$ pattern (Equation (10)) dielectric constant increases following the explicit critical-like scaling [61].

**Figure 5:**
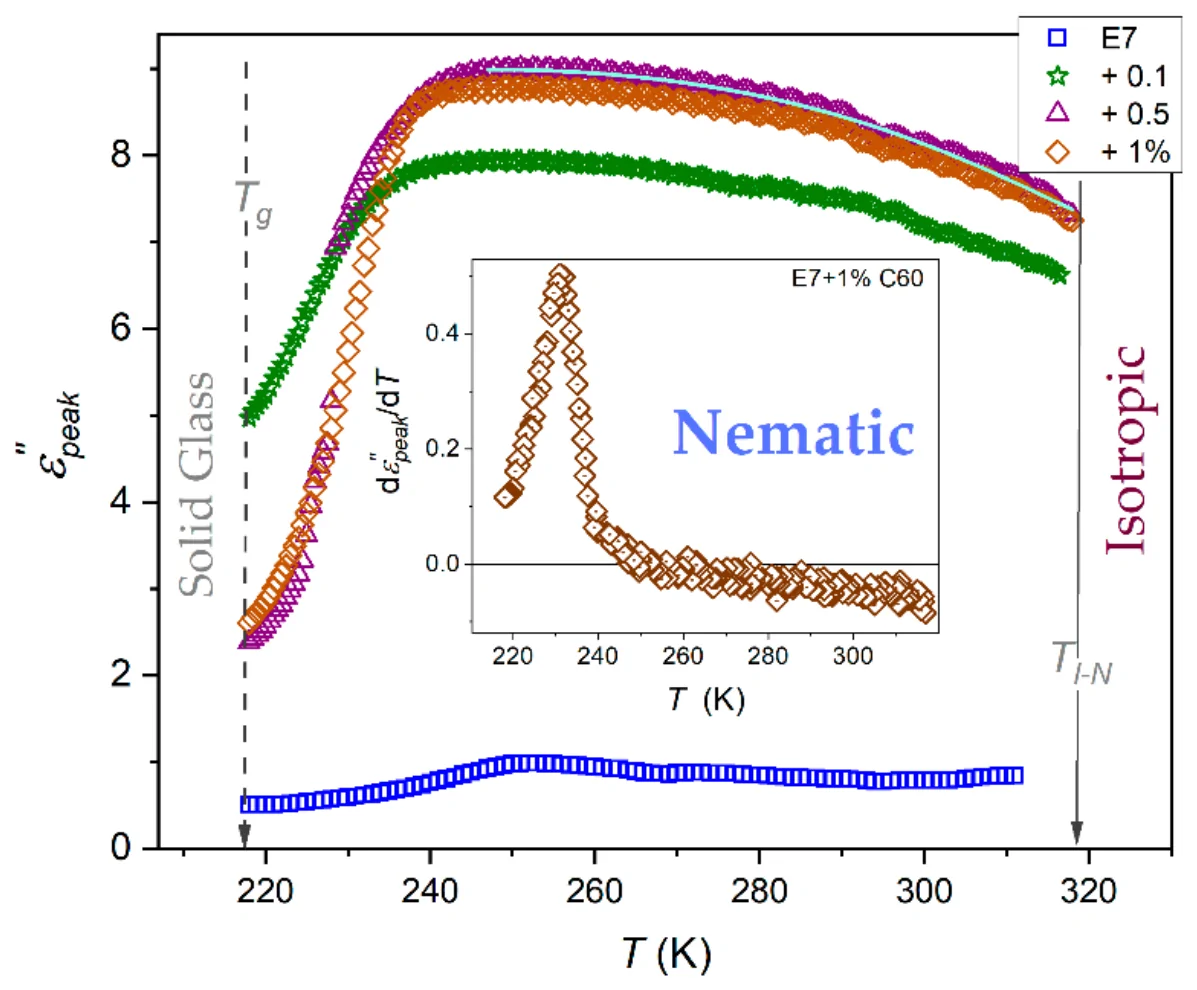
Temperature changes in the primary loss curve maximum in E7 and related nanocolloids with C_60_ fullerene nanoparticles. The scaling of data in the nematic phase for x=0.5% is related to the parallel of Equation (11), with the following parameters: $b_{1}=18.3$, $b_{2}=-14.8$, $b_{3}=5.2$, $b_{4}=-0.15$ and $T^{**}=334.2\;K$.

**Figure 6:**
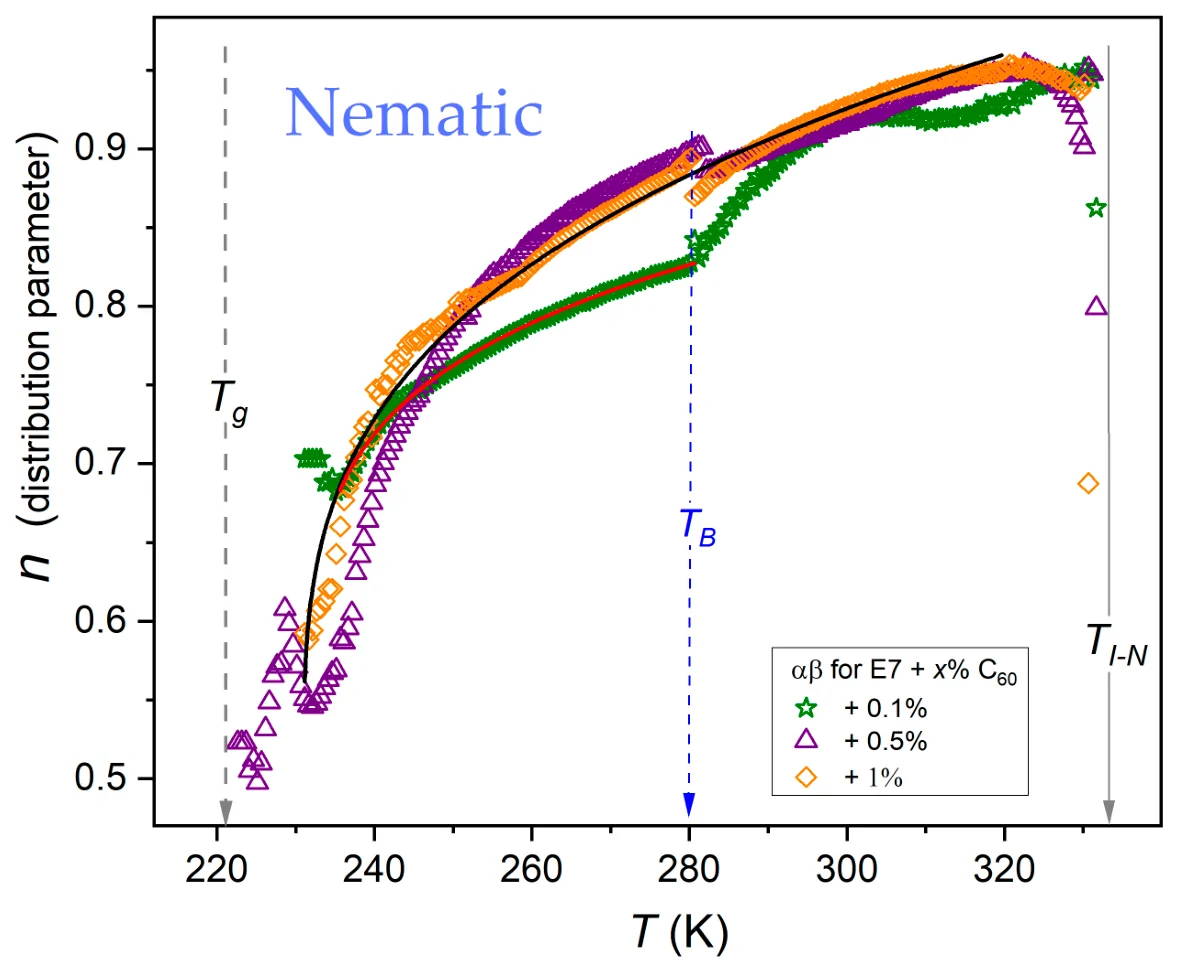
Changes in the high-frequency wing distribution parameter n for the primary relaxation time in the nematic phase of E7 and related nanocolloids with C_60_ fullerene nanoparticles. Note the changes at $T_{B}$, indicated by the dashed arrow in blue. $T_{B}$ in the plot can be correlated with the dynamic crossover temperature mentioned among ‘universalistic‘ features of the previtreous domain in the Section 1.

In the isotropic liquid phase of nematogens, the primary loss curve maximum ($\varepsilon_{peak}^{\prime \prime }\left(T\right)$) follows a pattern parallel to Equation (6) for the ‘critical’ anomaly of dielectric constant $\varepsilon \left(T\right)$ [61,66,67]. Both anomalies are governed by the volume occupied by prenematic fluctuations. For dielectric constant, they are detected by the decrease in dielectric constant due to the cancellation of the contribution from permanent dipole moments within prenematic fluctuations. The pretransitional anomaly of $\varepsilon_{peak}^{\prime \prime }\left(T\right)$ detects fluctuations via the energy loss impact required for molecular reorientation. Figure 5 shows changes of $\varepsilon_{peak}^{\prime \prime }\left(f\right)\left(T\right)$ in the nematic phase of tested nanocoloids: the pattern of changes also resembles the pattern for the dielectric constant presented in Figure 3. Such scaling is explicitly shown in Figure 5. $\varepsilon_{peak}^{\prime \prime }\left(T\right)$ changes in the immediate vicinity of $T_{g}$ are even stronger and more characteristic than for the dielectric constant, as shown by the derivative-based plot in the inset in Figure 5. The evidence presented in ref. [61], where nanocolloids E7 + BaTiO_3_ (2r=50 nm, paraelectric) nanoparticles and the results presented above for nanocolloids E7 + C_60_ fullerenes (2r=0.7 nm) can be considered a significant argument for pretransitional (previtreous) effect on $T{\to T}_{g}$ with the critical-like characterization. It is further supported by the critical-like scaling pattern for the parameter n describing the high-frequency part of the distribution of the dominant (*primary, alpha*) relaxation time, shown in Figure 6:

(12) $$n\left(T\right)\approx n_{ref.}+{\left(T-T_{g}\right)}^{\theta }$$

with $n_{ref.}=0.5\pm 0.05$, and θ=0.5±0.1.

Notably, a parallel relation was evidenced for the isotropic liquid phase of octyloxycyanobiphenyl (8OCB) and its nanocolloids with BaTiO_3_ nanoparticles [61].

**Figure 7:**
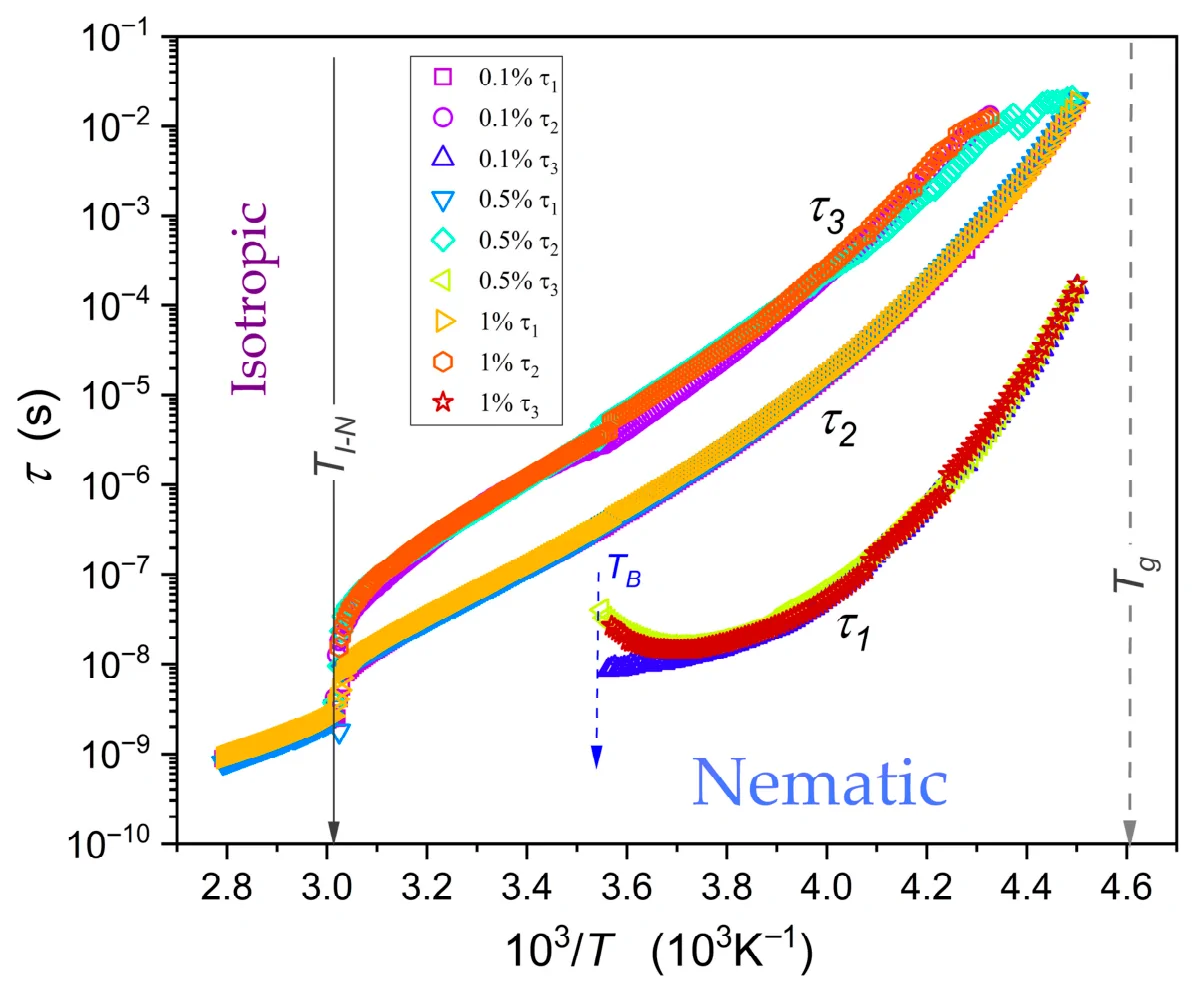
The Arrhenius scale plot for relaxation times in the isotropic and nematic phases of E7 and related nanocolloids with C_60_ fullerene nanoparticles, with concentrations shown in the figure. The indicated dependences relate to three relaxation ($\tau_{i}$) processes observed in BDS spectra. $T_{B}$ can be correlated with the dynamic crossover temperature mentioned among ‘universalistic‘ features of the previtreous domain in the Section 1. Note: all processes are non-Arrhenius as indicated by non-linear patterns of changes.

The map of relaxation time in E7 + C_60_ fullerene nanocolloids is shown in Figure 7. The single dominant primary relaxation time in the isotropic liquid phase relaxation continues in the nematic phase. However, in the LC mesophase additional relaxation processes emerge. Below $T_{B}\sim 280\;K$ the third—and much faster—relaxation time can be detected. All processes follow explicitly non-Arrhenius (SA) patterns, which is validated by nonlinear changes in Figure 7, prepared using the Arrhenius scale: lnτ or ${log}_{10}\tau$ vs. 1/T.

Such behavior is commonly scaled by the Vogel–Fulcher–Tammann (VFT) equation, which is the practical replacement for the general super-Arrhenius (SA) equation with the apparent, temperature-dependent activation energy $E_{a}\left(T\right)$ [14,15,16,17,18]:

(13) $$\tau \left(T\right)=\tau_{\infty }exp\left(\frac{E_{a}\left(T\right)}{RT}\right)\;\;\;\Rightarrow \;\;\;\tau \left(T\right)=\tau_{\infty }exp\left(\frac{E}{T-T_{0}}\right)=\tau_{\infty }exp\left(\frac{D_{T}T_{0}}{T-T_{0}}\right)$$

where the left part is for the general SA relation and the right one is for the VFT counterpart, with $E_{a}=ER/t={RD}_{T}T_{0}/\left[\left(T-T_{0}\right)/T\right]$, E=const, $D_{T}$ is the fragility strength parameter, $T_{0}<T_{g}$ is the extrapolated VFT singular temperature; R is for the gas constant.

The VFT equation has become so popular that it is often considered a heuristic ‘universal’ pattern for general previtreous dynamics [14,15,16,17,18]. The enormous success of the VFT relation results from its ’functional flexibility’ and simple application to empirical data. This portrayal was also successfully applied to pure E7 and E7 in nano-sieves [25,26].

However, recent in-depth analyses have explicitly shown that, in glass-forming systems, the VFT equation is only an effective descriptive tool [17]. Analysis of a set of model equations for scaling previtreous dynamics showed an explicit preference for only two scaling dependencies.

The first is the MYEGA equation, which can be obtained by introducing $E_{a}\left(T\right)=RKexp\left(C/T\right)$, K,C=const, to the basic SA equation [17,73,74]. It was successfully implemented for testing the previtreous dynamics in E7 + BaTiO_3_ NPs nanocolloids [61].

The second equation, composed of the ‘*Critical*’ and ‘*Activated*’ terms, has been derived by Drozd-Rzoska [17,75]:

(14) $$\tau \left(T\right)=C_{\Gamma }{\left(\frac{T-T_{g}^{*}}{T}\right)}^{-\Gamma }{\left[\exp\left(\frac{T-T_{g}^{*}}{T}\right)\right]}^{\Gamma }=C_{\Gamma }{\left(t^{-1}\exp t\right)}^{\Gamma }$$

where $t=\left(T-T_{g}^{*}\right)/T$ and $T_{g}^{*}<T_{g}$ is the extrapolated singular temperature.

The power exponent in Equation (14) can be expressed via basic empirical metrics of the glass transition:

$\Gamma =m\ln10\left(T_{g}/T_{g}^{*}\right)/\left(1/\left(\Delta T_{g}^{*}/T_{g}\right)-1\right)$, $\Delta T_{g}^{*}=T_{g}-T_{g}^{*}$ and m is the fragility metric of a glass-forming system. The value of the exponent Γ determines their relative share in the previtreous effect [75].

For systems composed of molecules with uniaxial symmetry, which is also the case of E7-based systems, the critical-like contribution dominates and a fair portrayal can be obtained even via a single critical-type term [17,75,76,77]:

(15) $$\tau \left(T\right)=C{\left(T-T_{g}^{*}\right)}^{\Gamma^{\prime }}$$

Equation (14) results from the empirical ‘universalistic’ finding for the ‘steepness index’ s(T) or alternatively apparent activation enthalpy $H_{a}\left(T\right)$ or the apparent fragility $m_{P}\left(T\right)$ previtreous behavior [17,75]:

(16) $$s\left(T\right),m_{P}\left(T\right),H_{a}\left(T\right)=\frac{A}{T-T_{g}^{*}}\;\;\;\Rightarrow \;\;{\left[s\left(T\right),m_{P}\left(T\right),H_{a}\left(T\right)\right]}^{-1}=A^{-1}T-A^{-1}T_{g}^{*}=aT-b\;\;\;\;$$

where the left-hand-related magnitudes are associated with the following transformation of $\tau \left(T\right)$ empirical data [17,75]:

(17) $$\tau \left(T\right)\;\;\;\Rightarrow \;\;\;\;s\left(T\right)=\frac{d\ln\tau \left(T\right)}{d\left(1/T\right)}=\frac{H_{a}\left(T\right)}{R}=\left(T_{g}ln10\right)m_{P}\left(T\right)$$

where $m_{P}\left(T\right)=\left(T_{g}/{log}_{10}e\right)\left(d{log}_{10}\tau \left(T\right)/d\left(T_{g}/T\right)\right)$ is the apparent fragility and the fragility metric $m=m_{P}\left(T_{g}\right)$; $m_{P}\left(T\right)$ is the steepness index for the normalized Arrhenius plot ${log}_{10}\tau$ vs. $T_{g}/T$, known as the Angell plot.

**Figure 8:**
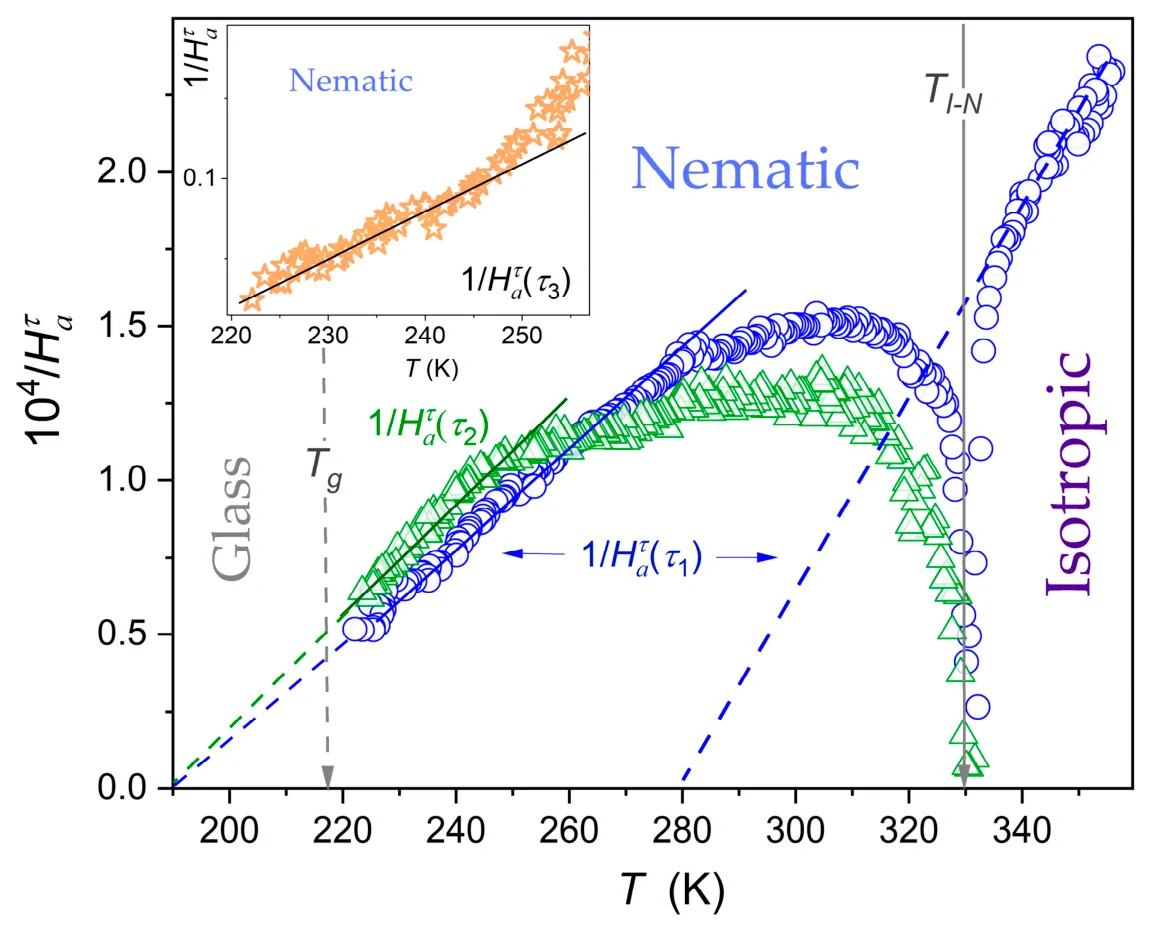
Reciprocals of the apparent activation enthalpy-related steepness index in the nematic phase of E7 and related nanocolloids with C_60_ fullerene nanoparticles (x=0.5%). The linear behavior is consistent with Equation (16). The plot relates to three relaxation times, as shown in Figure 7. The extrapolated singularity for the nematic phase is related to $T_{g}^{**}\left(nem.\right)\approx 190\;K$ and for the isotropic liquid phase: $T_{g}^{**}\left(iso.\right)\approx T_{B}^{**}=280\;K$. The analysis is for relaxation times $\tau_{i}$ indicated in Figure 7.

Figure 8 shows the evolution of the apparent activation enthalpy (alternatively: apparent fragility or the steepness index) of E7 + C_60_ fullerene nanocolloids, showing the ‘universalistic’ behavior defined by Equation (16). Notable is the strong impact of the I-N transitions vicinity, which can be associated only with the impact of pretransitional fluctuations associated with the weakly discontinuous near-critical nature of this phase transition. We stress this issue because the evidence for the influence of critical fluctuations on the primary relaxation time has been poorly documented to date.

Notable is the appearance of the behavior defined by Equation (16) also in the isotropic liquid phase, with the singularity at $T_{iso}^{*}\approx 280\;K$. For such functional dependence it can be associated only with the Mode Coupling Theory (MCT) singular behavior [17,62], predicted for so-called ergodic high-temperature dynamic domain in glass-forming systems expected for the time scale $\tau \left(T_{B}\right)\gg 10^{-7\pm 1\;}s$ [17,62]. Hallmarks of the crossover at $T_{B}\approx 280\;K$ are also visible for other properties discussed in this section.

**Figure 9:**
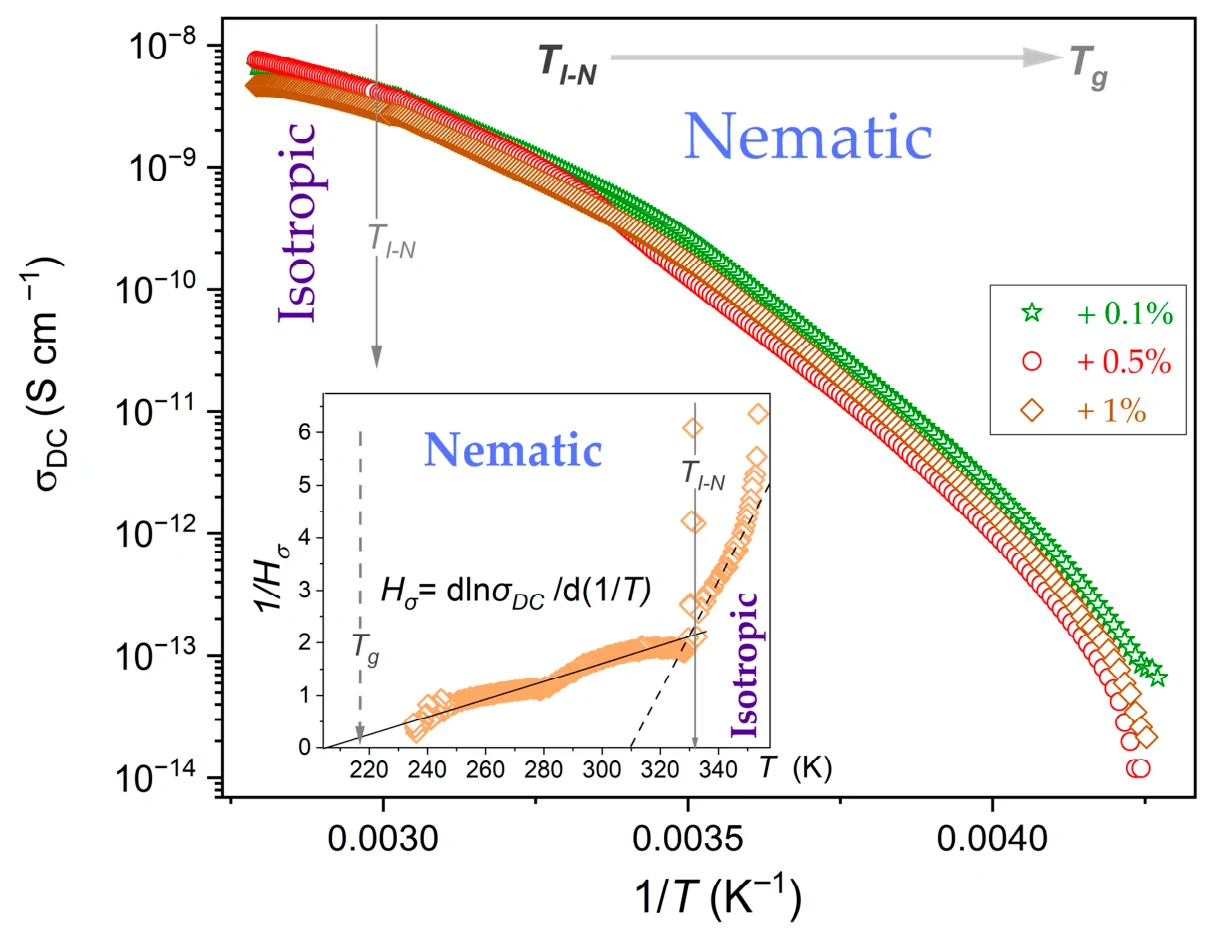
Changes in DC electric conductivity in the nematic phase of E7 and related nanocolloids with C60 fullerene nanoparticles. The inset shows the pattern of steepness-index (apparent activation enthalpy) scaling; note the links to Equation (18).

Figure 9 presents the behavior of the DC electric conductivity in the nematic and isotropic phases of tested nanocolloids. The inset in Figure 9 shows the related apparent enthalpy, scaled via the counterpart of Equation (16) derived in Equation [75], namely:

(18) $$\sigma \left(T\right)=C_{\Gamma }{\left(t^{-1}\exp t\right)}^{-\Gamma^{\prime }}\Leftrightarrow \;\frac{d{ln\sigma }^{-1}}{d\left(1/T\right)}=H_{\sigma }\left(T\right)=\frac{S}{T-T_{g}^{*}}$$

Specific universal feature of the previtreous dynamics in glass-forming molecular liquids, extending even up to $\sim T_{g}+$150 K, is the translational–orientational decoupling. It is expressed by the fractional Debye–Stokes–Einstein (DSE) law linking DC electric conductivity ($\sigma_{DC},\sigma$) and the primary relaxation time [78]:

(19) $$\sigma \left(T\right)\times {\left[\tau \left(T\right)\right]}^{F}=C=const\;\;\;\;\Rightarrow \;\;\;\;\;\;log\sigma \left(T\right)=C-F\times log\tau \left(T\right)$$

where F is the fractional exponent, showing the degree of the translational–orientational decoupling.

F=1 is for the standard DSE law, when translational processes are in time with orientational ones. The explicit fractional DSE behavior is for delayed or speed-up orientational processes in comparison to translational ones, related to F<1 and F>1, respectively. Generally, in glass-forming molecular liquids, the standard pattern is evidenced by F=1 for $T>T_{B}$ (high-temperature dynamic domain) and F<1 for $T_{B}>T>T_{g}$ (low-temperature dynamic domain).

**Figure 10:**
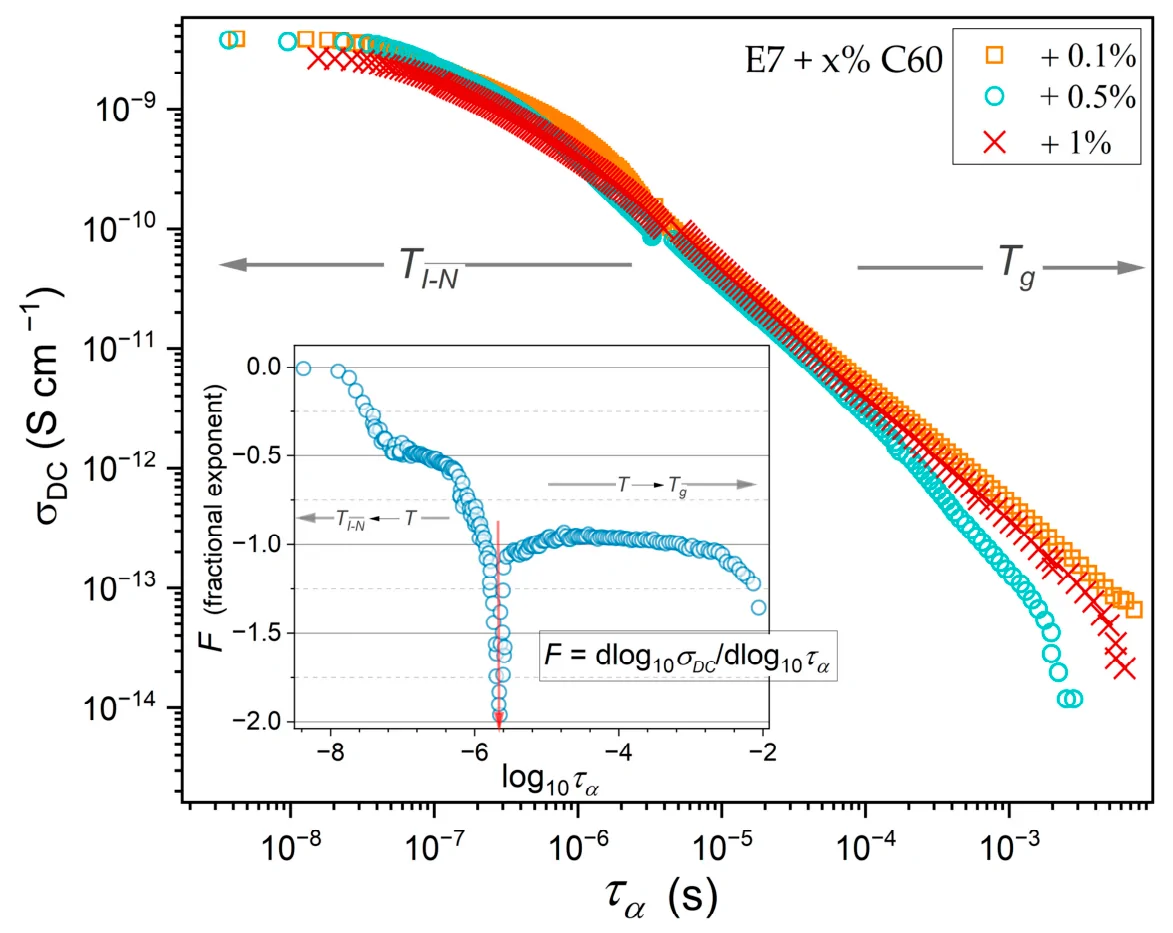
Fractional DSE translational–orientational coupling test (Equations (19) and (20)), tested via the presentation in the log–log scale for E7 + C_60_ fullerene nanocolloids. The derivative analysis in the inset yields changes in the fractional exponent *F*. The red vertical arrow indicates the *F*-singularity associated with $\tau_{\alpha }=2.5$ μs.

Figure 10 shows the F-DSE analysis for the tested nanocolloids, focusing on presentation using the left side of Equation (19). Notable is a non-standard nonlinear pattern in the broad surrounding of the I–N transition for $T>T_{B}$. However, as the amount of C_60_ fullerene nanoparticles increases, the rising nonlinearity appears as the glass transition is approached. The direct insight into crucial testing for the pattern of translational–orientational coupling/decoupling exponent F enables the transformation of empirical data via the following equation [78]:

(20) $$F=-\frac{log\sigma \left(T\right)}{log\tau \left(T\right)}$$

Its implementation is shown in the inset in Figure 10, revealing two domains that can be explicitly linked to the high- and low-temperature dynamical domains discussed above.

There is also an explicit ‘anomaly’ indicated by the vertical red arrow for the time scale coupled to the temperature $T_{B}\approx 280\;K$, noted above. Such an ‘anomaly’ has to be linked to the impact of fullerene nanoparticles, since it is absent for ‘pure’ E7 [55]. For times scale $\tau <\tau_{B}=\tau \left(T_{B}\right)$ there is a systematic drop of the exponent F, until it reaches F≈1 for some part of the low-temperature dynamic domain $\tau >\tau_{B}$. Near $T_{g}$ the decoupling appears again, but in this case it is associated with F>1.

## 4. Discussion

### 4.1. I-N Transition: Critical Behavior and Glassy Dynamics

#### 4.1.1. I-N Transition: Critical Behavior

I-N transition is included in the canon of *Critical Phenomena Physics*, as the model case of a weakly discontinuous transition is inherently associated with long-range pretransitional effects driven by multimolecular fluctuations [7,8,72]. It shows the symmetry of the neighboring phases, i.e., they are pre-nematic in the isotropic liquid and pre-isotropic in the nematic phase. This is particularly evident in heat capacity $C_{P}\left(T\right)$ studies or in the order parameter S(T) tests, in the nematic phase. The most classic are pretransitional effects related to the Cotton–Mouton Effect, Rayleigh Light Scattering ($R_{LS}$), or Kerr Effect (KE), for which the exceptional common pretransitional pattern inspired the Landau–de Gennes (LdG) model [7,8] development—one of the most important phenomenological concepts in *liquid crystal*s and *soft matter physics*, namely [66,67]:

(21) $$KE,R_{Ls},CME\propto \frac{1}{T-T^{*}}$$

To derive such behavior, De Gennes developed Landau’s expansion of the free energy with respect to the local order parameter to $C_{P}\left(T\right)$ or S(T) behavior [66,67] as well.

KE and CME describe optical birefringence due to the strong electric or magnetic fields impacts, namely $\Delta n/E^{2}$ and $\Delta n/H^{2}$, respectively. The above methods directly detect pretransitional/critical-like fluctuation. They employ light, so the observation time scale is: $t_{obs.}=1/f_{ligth}\ll \tau_{fluct.}$ Later, the same pattern was noted for Nonlinear Dielectric Effect (NDE), detecting changes in dielectric constant under the strong electric filed ${\Delta \varepsilon }^{E}/E^{2}$. However, for NDE: $t_{obs.}>\tau_{fluct.}$ [8], since it employs radio-frequency detection in the kHz-MHz range. The inclusion of NDE in research has led to finding a set of discrepancies between experimental results and the Landau–de Gennes model. This puzzle was only recently solved by the new model proposed by Drozd-Rzoska [8]. It also enabled, for the first time, a model-based derivation of Equation (6) describing pretransitional changes in the dielectric constant.

In ref. [8], special attention was paid to the ‘Contrast Factor’ (CF) in studies of pretransitional effects. Namely, fluctuations–‘heterogeneities’ should differ significantly from their local environment with respect to the given research method. An example can be the absence of a pretransition anomaly for the dielectric constant in the isotropic phase of nematogens with a permanent dipole moment perpendicular to the long axis of the molecule, where the lack of $\varepsilon \left(T\right)$ pretransitional anomaly is related to CF ≈ 0 [8,60]. For LC molecules with the permanent dipole moment parallel to the long molecular axis CF≫ 0, which leads to a significant pretransitional effect, is shown in the Section 3.

The anomaly is even more pronounced for $\varepsilon_{peak}^{\prime \prime }\left(T\right)$, since the energy loss required for the reorientation is essentially different with fluctuations in comparison to their surroundings.

Notably, NDE defined as ${\Delta \varepsilon }^{E}/E^{2}=\left(\varepsilon -\varepsilon \left(E\right)\right)/E^{2}$ detects directly pretransitional fluctuations, i.e., ‘fluctuations’/ ‘heterogeneities in ‘different’ surrounding [8].

Dielectric constant changes $\varepsilon \left(T\right)$ detects pretransitional changes ‘indirectly’, i.e., via the impact on the volume they occupy in the sample.

5CB is one of E7 mixture components. In refs. [70,71] it has been shown that the impact of prenematic fluctuations in the isotropic liquid phase tested via the range of $\varepsilon \left(T\right)$ critical anomaly (Equation (6)) can extend even up to $\sim T_{I-N}+100\;K$ in the isotropic liquid phase, for phase $T>T_{I-N}$. In the nematic phase, for $T<T_{I-N}$, the critical-like behavior extends down to $\sim T_{I-N}-80\;K$, as shown in Figure 3 by the critical-like portrayal via Equations (10) and (11). This was possible only due to the unique endogenic ordering of LC molecules induced by C_60_ fullerene nanoparticles in the nematic phase.

#### 4.1.2. I-N Transition: Glassy Dynamics

A quarter of a century ago, Letz et al. [79] and Theenhas et al. [80] modeled the hard ellipsoid fluid using a mean-field mode-coupling approach. They showed that this is a unique model ‘glassy’ system, exhibiting uniaxial fluctuations/heterogeneities and two basic relaxation processes. The first one relates to collective processes (‘fluctuations’) and the four-point (4) correlation function. The second one concerns single-particle processes and the two-point (2) correlation function. Both are described by the same functional dependence, characteristic of the MCT approach in the previtreous domain [79,80]:

(22) $$\tau_{4,2}\propto {\left(T-T_{MCT}^{4,2}\right)}^{-\phi }$$

where $T_{MCT}^{4}$ and $T_{MCT}^{2}$ are extrapolated singular MCT temperatures, coupled to $\tau_{4}\left(T\right)$ and $\tau_{2}\left(T\right)$, respectively.

However, the collective relaxation time (4-point correlation function) singularity $T_{MCT}^{4}$ is located just below the solidification temperature $T_{S}$ and single element related $T_{MCT}^{2}$ ca. 30–40 K below $T_{S}$. Such behavioral scenarios for dynamics fairly coincides with the behavior observed in the isotropic liquid phase of nematogens for NDE-detected relaxation time and the primary relaxation time (*alpha*) from BDS tests, as can be noted from the given report and refs. [76,81,82,83]. Hence, $T_{MCT}^{4}$ correlates with $T^{*}$ and then $\tau_{4,2}{\to \tau }_{fluct.}$. for two-point related relaxation $\tau_{2}\left(T\right)\to \left(\tau_{\alpha },\tau \right)$ and $T_{MCT}^{2}$ $\to T_{B}$ [66,67,81,82,83]. Indeed, such behavior was validated by Drozd-Rzoska et al. [8,57,58,59,60,61], in the isotropic phase of numerous LC materials, with a clear prevalence over the often used Vogel–Fulcher–Tammann (VFT) equation. Notably, the *Critical and Activated* Equation (14) [75] fairly approximated the MCT critical-like equation $\tau_{2}\left(T\right)\propto {\left(T-T_{MCT}^{2}\right)}^{-\phi }$, well above the singular temperature [15,74].

The above behavior for $\tau_{2}\left(T\right)$ (i.e., $\tau_{\alpha }\left(T\right)$) and $\tau_{4}\left(T\right)$ (i.e., $\tau_{fluct.}\left(T\right)$) was confirmed in BDS and NDE(f) spectroscopic studies in the isotropic phase of nematogens [8,65,66,67,71,76,77]. Notable can also be Transient Grating Optical Kerr Effect (TG OKE) studies, with the extreme time-scale insight that explicitly yields evidence for $\tau_{2}\left(T\right)$ and $\tau_{4}\left(T\right)$ is possible using single scans for subsequent temperatures [81,82,83].

### 4.2. Glass Transition: Glassy Dynamics and the Critical Behavior

It is generally believed that the glass transition is a specific ‘dynamic’ phenomenon, where universalistic or singular features are restricted to dynamic properties, as indicated in the Section 1. In glass-forming systems, this behavior is most often scaled by the VFT equation; however, it has only an effective meaning, as evidenced recently [15]. The optimal description for different types of glass formers is given by Equation (14), and for systems with the dominant uniaxial molecular symmetry and also the explicit critical-like portrayal [17,71]:

(23) $$\tau_{2}\left(T\right)\propto {\left(T-T_{g}^{*}\right)}^{-\varphi }\;\;\;\;\;\;\;\;\;\mathrm{and}\;\sigma_{DC}\left(T\right)\propto {\left(T-T_{g}^{*}\right)}^{-\varphi_{1}}$$

Among the dominant universalistic dynamic properties in the immediate previtreous area, particularly significant is the fractional translational–orientational decoupling, particularly for time scales $t>t_{B}\sim 10^{-7\pm 1}$ s (low-temperature dynamic domain). Generally, it is linked to constant fractional exponent in the subsequent dynamic domain, and the near-smooth crossover: F≈1→ F<1 (see Equations (19) and (20)). For E7 + C_60_ fullerene nanocolloids, a more complex picture appears, with increasing decoupling (exponent F) in the immediate pretransitional/previtreous region, as shown in the inset of Figure 10.

Two decades ago, Nielsen et al. [84] empirically pointed out that the parameter describing the high-frequency distribution of low-molecular weight glass distribution of the primary relaxation time can be universal: $n\left(T{\to T}_{g}\right)\to \sim 1/2$. This report confirms this finding, and also shows explicit critical-type behavior (Equation (12)) for n(T) extended even up to 60 K above $T_{g}$. It is worth mentioning that such a critical characteristic of n(T) was also observed in the isotropic liquid phase of 8OCB, which is one of E7 components.

To reach a breakthrough in understanding the nature of the glass transition, reliable scaling relations describing previtreous/pretransitional changes are particularly important. For this insight, methods directly detecting multimolecular heterogeneities are essential [85,86,87,88,89]: perhaps counterparts of critical fluctuations. These could be TG OKE [81,82,83], time-frequency-resolved NDE [85], or time-resolved EKE, but they have been tested essentially only in the isotropic phase of nematogens, which is often considered a model case for the glass transition. This results from the ratio between single-molecule-related and collective relaxation times, namely: $\tau_{4}/\tau_{42}\;\sim \tau_{fluct.}/\tau_{\alpha }=10^{3}-10^{4}$ [60,61,62,81,82,83]. For the I-N transition $\tau =\tau_{\alpha }=10^{-9}-10^{-8}$ [57,58,59,60,61,66,67] and then the detection by mentioned heterogeneities/fluctuations coupled methods it is possible in a wide range of temperatures. For the glass transition $\tau_{\alpha }\left(T_{g}\right)\approx 100\;s$, and then the $\tau_{fluct.}\left(T_{g}\right)\sim 10^{6}$ s—which makes experimental tests impossible in practice. Nevertheless, the ‘operational window’ for such tests can open slightly remote $\tau_{\alpha }\left(T_{g}\right)$, as shown in time-resolved NDE studies [85,86]. However, direct studies of multimolecular heterogeneities are still primarily tested for detecting their presence [87,88], without attempts, or even the possibility, to determine temperature scaling.

In such a context, the pretransitional changes shown in this work and ref. [61], for ε(T) and $\varepsilon_{peak}^{\prime \prime }(T)$ can be particularly significant as they reduce the problem related to the time scale of collective process. Notably, such evidence was also recently reported for standard glass-forming molecular liquids, particularly composed of molecules with dominant uniaxial symmetry [89]. Of particular importance here is the critical-like scaling of the relaxation time distribution parameter. To complete this picture, one can also recall recent evidence of critical-like scaling in the configurational entropy or the related contribution to the heat capacity for standard glass formers [90].

## 5. Conclusions

This work presents results from studies of nematogenic LC E7 mixture + C_60_ fullerene nanoparticle nanocolloids, in bulk samples and using BDS scans to obtain insights beyond the standard patterns reported so far. This led to finding of several new properties that may have implications for both fundamental modeling and applications. Both of these issues are addressed by demonstrating that even small amounts of C_60_ fullerene nanoparticles can induce a stable endogenous arrangement of rod-like molecules, a phenomenon previously achieved in bulk samples using strong external fields (magnetic or electric).

Another significant result of this work may have significant implications for glass transition physics. First, it is one of the few studies to date demonstrating the endogenous influence of nanoparticles on the previtreous properties of glass-forming systems. Second, it demonstrates the coexistence of coherent dual glassy and critical-like features in both the I-N transition and the glass transitions—potentially important experimental evidence in the still-unresolved debate on the nature of the glass transition. In this context, it is also worth recalling the authors’ recent work on E7 + BaTiO_3_ nanoparticle nanocolloids.

The authors also want to draw attention to new, previously unreported critical-like changes upon approaching the glass temperature, as well as to a number of similarities emerging for pretransition effects near the I-N transition and previtreous changes.

This report and ref. [61] indicate a significant and even decisive influence of nanoparticles on the properties of the supercooling E7 nematogenic LC matrix. The type and size of the nanoparticles are important here, as are the significant influences of multimolecular pre-transition fluctuations, whose size for the I-N transition changes from the microscale near the phase transition to the nanoscale with distance.

The results of this work and ref. [61] also indicate the significant role of nanoparticle-induced ‘frustration’ on previtreous properties, which may have significant implications for further research into this great cognitive challenge. This research direction remains underexplored. However, the influence of nanoparticles is particularly pronounced in some systems, such as LC materials, where strong interactions occur.

The specific features of LC-based nanocolloids, particularly glass-forming E7, require further studies aimed at a microscopic explanation, including supplementary evidence from optical/microscopic observations that also match the additional impact of the electric field on orientation-related behavior.

## Data Availability

The data presented in this study have been deposited in Figshare and are currently under embargo, as the associated manuscript is under review. The dataset is available to editors and reviewers through a private access link (below). The data will be made publicly available upon completion of the related research activities and publication of the corresponding article. Private link: https://figshare.com/s/06b31a6f39f2ebe1e47b.
